# Validity and reliability of a portable gait analysis system for measuring spatiotemporal gait characteristics: comparison to an instrumented treadmill

**DOI:** 10.1186/s12984-016-0115-z

**Published:** 2016-01-20

**Authors:** Lars Donath, Oliver Faude, Eric Lichtenstein, Corina Nüesch, Annegret Mündermann

**Affiliations:** Institute of Exercise and Health Sciences, University of Basel, Birsstrasse 320 B, 4052 Basel, Switzerland; Clinic for Orthopaedics and Traumatology, University Hospital Basel, Spitalstrasse 21, 4031 Basel, Switzerland; Division of Sport Science, University of Konstanz, Universitätsstrasse 10, 78457 Konstanz, Germany

**Keywords:** Body worn sensors, Spatiotemporal gait characteristics, Accelerometers, Gyroscope, Validity, Reliability

## Abstract

**Background:**

Gait analysis serves as an important tool for clinicians and other health professionals to assess gait patterns related to functional limitations due to neurological or orthopedic conditions. The purpose of this study was to assess the validity of a body-worn inertial sensor system (RehaGait®) for measuring spatiotemporal gait characteristics compared to a stationary treadmill (Zebris) and the reliability of both systems at different walking speeds and slopes.

**Methods:**

Gait analysis was performed during treadmill walking at different speeds (habitual walking speed (normal speed); 15 % above normal walking speed; 15 % below normal walking speed) and slopes (0 % slope; 15 % slope) in 22 healthy participants twice 1 week apart. Walking speed, stride length, cadence and stride time were computed from the inertial sensor system and the stationary treadmill and compared using repeated measures analysis of variance. Effect sizes of differences between systems were assessed using Cohen’s d, and limits of agreement and systematic bias were computed.

**Results:**

The RehaGait® system slightly overestimated stride length (+2.7 %) and stride time (+0.8 %) and underestimate cadence (−1.5 %) with small effect sizes for all speeds and slopes (Cohen’s d ≤ 0.44) except slow speed at 15 % slope (Cohen’s d > 0.80). Walking speed obtained with the RehaGait® system closely matched the speed set on the treadmill tachometer. Intraclass correlation coefficients (ICC) were excellent for speed, cadence and stride time and for stride length at normal and fast speed at 0 % slope (ICC: .91–1.00). Good ICC values were found for stride length at slow speed at 0 % slope and all speeds at 15 % slope (ICC: .73–.90). Both devices had excellent reliability for most gait characteristics (ICC: .91–1.00) except good reliability for the RehaGait® for stride length at normal and fast speed at 0 % slope and at slow speed at 15 % slope (ICC: .80–.87).

**Conclusions:**

Larger limits of agreement for walking at 15 % slope suggests that uphill walking may influence the reliability of the RehaGait® system. The RehaGait® is a valid and reliable tool for measuring spatiotemporal gait characteristics during level and inclined treadmill walking.

## Background

Gait analysis serves as an important tool for clinicians and other health professionals to assess gait patterns related to functional limitations due to neurological or orthopedic conditions [1]. For instance, assessing and interpreting changes in spatiotemporal gait characteristics (e.g. gait speed, stride time, stride length, stride width, stride variability) has become important for predicting fall risk [2, 3]. These studies have been mainly conducted using stationary treadmills or portable optometric systems and gait mats [4]. However, this analysis is time-consuming and/or costly and obtained laboratory data may not be completely transferable to free-living conditions. For instance, elderly persons tend to walk at faster average speeds when walking longer (>20 m) than shorter distances (<10 m) [5] and parameters such as gait variability are more reliable when assessed for walking distances of at least 20 m [6, 7]. Hence, walking should be assessed outside the laboratory or on treadmills where persons can walk for extended periods of time. Moreover, simpler and low-cost mobile systems are desirable for evaluating pathological gait patterns outside the laboratory for treatment planning and evaluation.

Although body-worn sensor systems facilitate data collection outside the laboratory or clinic, most tests are usually performed in controlled settings. These laboratory or clinical environments for assessing gait are characterized by a level walkway, and patients are typically asked to walk at their habitual walking speed (normal speed). In contrast, free-living conditions include walking on varying slopes and surfaces and at different walking speeds resulting in locomotor adaptations including altered limb positioning and acceleration [8, 9]. To date, the effects of these changing environmental factors on the validity and reliability of body-worn sensor systems are not fully understood. Validity and reliability studies of gait analysis systems have to trade off the controlled setting of treadmill walking where gait parameters of many consecutive steps at predefined environmental conditions can be compared between systems and the real-time situation where gait parameters of only few isolated steps can be compared between different systems. Moreover, instrumented treadmills gait analysis is reliable [2] and considered a standard for evaluating the accuracy of portable and laboratory systems in measuring spatiotemporal gait characteristics in healthy subjects and patients [10–12].

In recent years, activity monitors for classifying physical activity and pedometers for counting the number of steps have become available. Most accelerometer-based systems only provide step counts, stride times or cadence. To date, systems precisely capturing spatiotemporal gait characteristics are mostly used in specific (research) settings [13]. Novel and more complex systems not only comprise accelerometers but also gyroscopes and magnetometers, and hence are able to compute spatiotemporal and specific kinematic parameters. Such systems have been used to explore specific pathological gait and balance patterns [13–15] and to evaluate training interventions [16, 17] in older adults and in patients with neurological diseases. RehaGait® (Hasomed, Magdeburg, Germany) is a commercial mobile system that specializes on measuring gait characteristics in field settings enabling gait data collection within less than 10 min (preparation: 1 min; calibration: 1 min; data collection: 1–8 min. The system comprises multi-directional accelerometers, gyroscopes and magnetometers, software computing spatiotemporal gait parameters as well as selected joint kinematics during overground or treadmill walking and running, a large database of normative gait data (more than 2000 healthy subjects ranging in age from 5 to 90 years) and an intuitive and comprehensive tablet user interface representing a promising tool for therapists. However, to date only limited data on the validity and reliability of RehaGait® are available [18]. Specifically, to date the validity of the RehaGait® for measuring gait characteristics of treadmill walking has not been determined. This information is critical for its potential application in research and clinical practice. Besides the parameters investigated here, the system also computes gait phases, foot to ground angle, foot height and hip circumduction.

The purpose of this study was to assess the validity of the body-worn sensor system RehaGait® for measuring spatiotemporal gait characteristics with a stationary treadmill and the reliability of both systems at different speeds and slopes.

## Methods

### Subjects

Twenty-two healthy participants (8 women, 14 men; age: 31.0 ± 3.7 years; height: 1.78 ± 0.11 m; body mass: 82.1 ± 23.4 kg; body mass index: 26.0 ± 7.0 kg/m^2^) participated in this study after providing informed written consent. This sample size provides a power of 95 % to detect large effects of agreement for the gait parameters assessed when considering an alpha-level of 5 % based on previous data [2]. Exclusion criteria were any medication intake; any orthopedic (e.g. low back pain, hip or knee endoprosthesis and ankle sprain), neurologic (hearing loss, equilibrium organ dysfunction) or internal diseases (hypertension, diabetes mellitus, heart disease and arterial occlusion); and any health impairments that may affect gait. The study was approved by the Ethikkomission Nordwestschweiz (Switzerland) and conducted in accordance with the declaration of Helsinki.

### Testing equipment

**Fig. 1:**
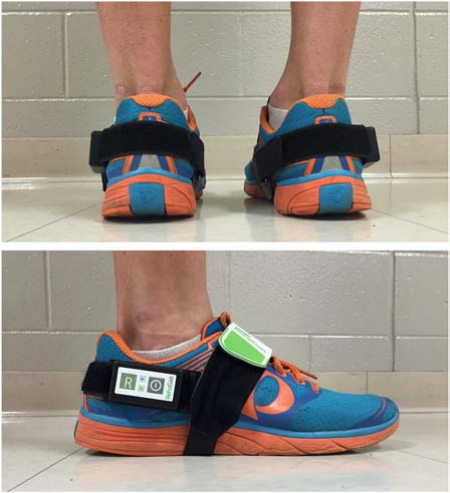
Photograph showing the senor placement on the lateral aspect of subjects’ personal shoes

Spatiotemporal gait data were collected simultaneously using the portable gait analysis system RehaGait® (Hasomed GmbH, Magdeburg, Germany) and an instrumented treadmill (Zebris FDM-T, Zebris medical GmbH, Isny, Germany). The RehaGait® system consists of two mobile inertial sensors (dimensions: 60 × 15 × 35 mm) and analysis software [19] and is a new version of the previously published RehaWatch® [18]. Each sensor comprises a 3-axis accelerometer (±16 g), a 3- axis gyroscope (±2000 °/s) and a 3-axis compass (±1.3 Gs). The sensors were attached to the lateral aspect of each shoe (Fig. 1) using special straps to measure linear acceleration, angular velocity and the magnetic field of the foot at a sampling rate of 500 Hz. Manufacturer proprietary software was used to obtain temporal and spatial gait characteristics.

The instrumented treadmill consists of a treadmill ergometer with an integrated pressure sensor mat comprising a matrix of high-quality capacitive force sensors (range, 1–120 N/cm^2^; precision, 1–120 N/cm^2^ ± 5 %) and analysis software. The walking surface of the treadmill (length: 1.5 m; width: 0.5 m) comprises 5378 force sensors. The system measures the dynamic pressure distribution under the feet while walking on the treadmill at a sampling rate of 120 Hz. Spatiotemporal gait characteristics were computed automatically from the pressure data within the software. According to the manufacturer, heel-strike is the time of initial contact (threshold, 1 N/cm^2^) and toe-off the last frame before all sensor pressure values for the foot of interest drop below the threshold. Stride length is calculated as the distance between two initial heel pressure points of alternate sides. Stride time is defined as the time between two consecutive heel-strikes of the same foot and cadence as the number of steps per minute. In addition, the treadmill speed set on the tachometer was recorded. We used the tachometer speed as reference value for the validity analysis and speed calculated from the pressure data for the reliability analysis.

### Testing procedure

Each participant was tested on two days (1 week apart) at the same time of the day wearing the same personal athletic shoes on both days. Habitual walking speed of each subject was determined in the first session as the average of three trials of 10-m normal overground walking assessed with photoelectric timing gates (Witty, Microgate, Bolzano, Italy) and termed normal speed. Participants were equipped with the RehaGait® and completed a familiarization trial of 5-min treadmill walking at normal walking speed in both sessions. All participants then performed six 5-min walking trials on the instrumented treadmill while wearing the RehaGait® device. Data for three walking speeds (normal walking speed; 15 % above normal walking speed; 15 % below normal walking speed [20, 21]) and two slopes were collected (0 % slope; 15 % slope [22, 23]; one 5-min trial per condition). Each trial was followed by a 5-min break between conditions. Testing order of these conditions was randomly assigned using a randomization table. Each 5-min trial consisted of at least 200 strides (double steps) [24].

### Data acquisition and analysis

For all experimental conditions, spatiotemporal gait characteristics were simultaneously recorded by the treadmill and the RehaGait® device. For both devices, walking speed (m/s), stride length (m), cadence (steps/min) and stride time (s) were recorded for each stride, and average values of 200 consecutive strides were computed for each session, participant and condition and used for further analysis. Data measured using both devices were used to determine validity. Data measured on the two separate days were used to determine the reliability of both systems.

### Statistical analysis

All statistical analyses were carried out in SPSS Version 22 (IBM Corporation, Amonk, NY). Separate 2 (device: RehaGait® vs. treadmill) × 3 (speed: normal vs. slow vs. fast) × 2 (slope: flat vs. inclined) × 2 (time: day 2 vs. day 1) fixed-factor linear mixed models were conducted for speed, stride length, cadence and stride time. Bonferroni post hoc tests were computed in case of occurring main effects (α = .01). Pairwise effect size estimation of the differences between both devices were calculated using Cohen’s d (trivial: d < 0.2; small: 0.2 ≤ d < 0.5; moderate: 0.5 ≤ d < 0.8; large d ≤ 0.8) as the standardized mean difference normalized to the pooled standard deviation for each condition. The agreement between RehaGait® and treadmill data and the test-retest reliability of data collected on two different days was analyzed for each parameter and condition by calculating the systematic bias (mean difference between devices/days) and root means square errors, and the limits of agreement (1.96*standard deviation of the difference between both devices/days) to obtain a 95 % random error component [25]. The limits of agreement indicate the range in which the difference of each two tests on the analyzed devices and days will fall with a probability of 95 % for each new individual. The results of this analysis are presented as Bland-Altman plots [26]. In addition, the intraclass correlation coefficients (ICC) with their 95 % confidence intervals were calculated using a two-way, random single measure analysis for each condition. Point estimates of the ICC were rated as excellent (0.9–1), good (0.73–0.9), moderate (0.4–0.74) and poor (0–0.39) [27].

## Results

### Validity

**Fig. 2:**
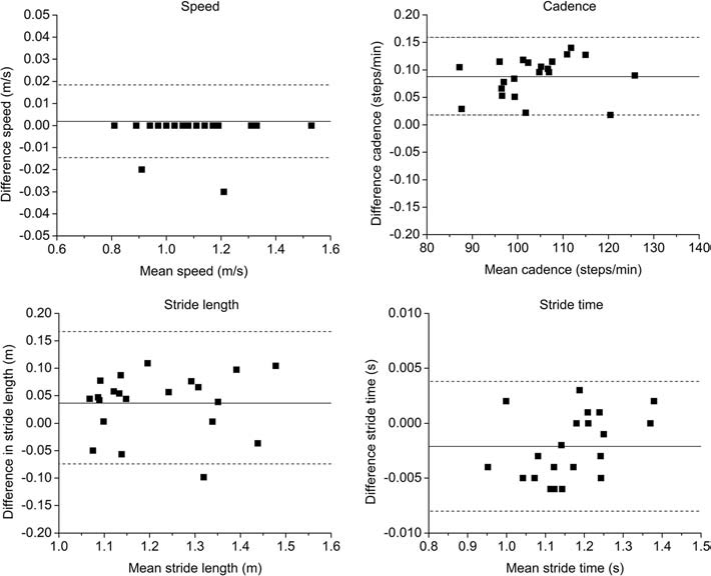
Bland-Altman plots for each gait characteristics for walking at normal speed at 0 % (*left*). Each graph presents the mean difference (*solid line*) and 1.96-fold standard deviation of difference (*dashed line*) indicating the limits of agreement between the RehaGait® and the instrumented treadmill

Independent of walking speed and slope, a main effect for the factor device was found for all gait characteristics (speed: *F* = 55.4, *P* < .001; stride length: *F* = 14.8, *P* = .002; cadence: *F* = 58.9, *P* < .001; stride time: *F* = 46.0, *P* < .001; Table 1). The RehaGait® system overestimated stride length and stride time and underestimate cadence with medium effect sizes for all speeds and slopes except for slow speed and 15 % slope (Table 2; Fig. 2). Walking speed obtained with the RehaGait® system closely matched the speed set on the treadmill tachometer. However, walking speed calculated from the pressure data underestimated tachometer speed at all speeds and slopes (Table 1). All effect sizes were small to moderate (Cohens’ d, 0.3–0.8). There were no significant device × speed or device × slope interactions. The limits of agreement for data measured with the RehaGait® and the instrumented treadmill were comparable at the two different slopes. ICC values of data measured using both devices for speed, stride length, cadence and stride time for each slope and walking are shown in Table 3. ICC values between devices were excellent for speed, cadence and stride time and for stride length at normal and fast speed at 0 % slope, and good for stride length at slow speed at 0 % slope and all speeds at 15 % slope. RMS errors of differences in gait parameters between the two systems for the different conditions ranged from 0.069 to 0.187 m/s (speed), 0.069–0.112 m (stride length), 0.085–0.102 steps/min (cadence) and 2.2–3.7 ms (step time) on day 1 (Table 4).

**Table 1:** Mean (one standard deviation) stride length, cadence and stride time measured at two different inclines (0 % slope, 15 % slope) and three different speeds (slow, normal, fast) using the RehaGait® and the instrumented treadmill

Condition	Tachometer speed (m/s)	Speed (m/s)		Stride length (m)		Cadence (steps/min)		Stride time (s)	
		RehaGait®	Treadmill	RehaGait®	Treadmill	RehaGait®	Treadmill	RehaGait®	Treadmill
*0 % slope*									
Slow speed	0.95 (0.16)	0.95 (0.16)	0.91^b^ (0.13)	1.148 (0.127)	1.118 (0.110)	96.7^a^ (8.7)	98.2 (11.2)	1.25 (0.11)	1.24 (0.13)
Normal speed	1.12 (0.19)	1.11^a^ (0.19)	1.04^b^ (0.17)	1.234^a^ (0.135)	1.188 (0.138)	103.8^a^ (9.6)	105.5 (12.4)	1.16^a^ (0.11)	1.15 (0.12)
Fast speed	1.28 (0.22)	1.29^a^ (0.23)	1.21^b^ (0.20)	1.331 (0.156)	1.319 (0.169)	109.8 (10.5)	110.6 (12.5)	1.11 (0.10)	1.10 (0.12)
*15 % slope*									
Slow speed	0.95 (0.16)	0.93 (0.14)	0.89^b^ (0.10)	1.195 (0.199)	1.157 (0.107)	94.3 (11.8)	94.7 (11.8)	1.29 (0.15)	1.29 (0.15)
Normal speed	1.12 (0.19)	1.12^a^ (0.20)	1.05^b^ (0.18)	1.310^a^ (0.184)	1.213 (0.135)	103.3 (13.3)	103.3 (13.0)	1.18 (0.14)	1.18 (0.13)
Fast speed	1.28 (0.22)	1.31^a^ (0.22)	1.21^b^ (0.21)	1.361 (0.150)	1.326 (0.137)	109.4 (11.8)	109.1 (11.8)	1.11 (0.11)	1.11 (0.11)

^a^significantly different from values measured with the instrumented treadmill (*P* < .01)

^b^significantly different from tachometer speed (*P* < .01)

**Table 2:** Effect sizes of the differences between both devices using Cohen’s d (trivial: d < 0.2; small: 0.2 ≤ d < 0.5; moderate: 0.5 ≤ d < 0.8; large d ≤ 0.8)

Condition	Speed	Stride length	Cadence	Stride time
*0 % slope*				
slow speed	0.25	0.15	0.01	0.01
normal speed	0.37	0.27	0.01	0.02
fast speed	0.37	0.03	0.07	0.01
*15 % slope*				
slow speed	0.85	0.80	0.88	0.74
normal speed	0.38	0.44	0.01	0.01
fast speed	0.40	0.25	0.02	0.02

**Table 3:** Intraclass correlation coefficients (ICC, two-way, random, single measure) with 95 % confidence interval for speed, stride length, cadence and stride time for each slope and walking

Condition	Speed	Stride length	Cadence	Stride time
*Validity*				
*0 % slope*				
Slow speed	.953 (.883–.981)	.897 (.746–.958)	1.000 (1.000–1.000)	1.000 (.999–1.000)
Normal speed	.985 (.964–.994)	.954 (.886–.981)	1.000 (1.000–1.000)	1.000 (1.000–1.000)
Fast speed	.944 (.863–.977)	.914 (.789–.965)	.975 (.937–.990)	1.000 (.999–1.000)
*15 % slope*				
Slow speed	.937 (.836–.976)	.763 (.417–.904)	1.000 (1.000–1.000)	1.000 (1.000–1.000)
Normal speed	.991 (.976–.996)	.727 (.327–.889)	1.000 (1.000–1.000)	1.000 (1.000–1.000)
Fast speed	.951 (.881–.980)	.833 (.597–.931)	.999 (.997–.999)	1.000 (1.000–1.000)
*Test–retest reliability RehaGait*®				
*0 % slope*				
Slow speed	.995 (.986–.998)	.968 (.913–.988)	.945 (.852–.979)	.941 (.843–.978)
Normal speed	.980 (.949–.992)	.804 (.506–.923)	.977 (.943–.991)	.981 (.951–.992)
Fast speed	.952 (.871–.982)	.833 (.552–.937)	.953 (.873–.982)	.972 (.925–.990)
*15 % slope*				
Slow speed	.999 (.997–1.000)	.866 (.651–.948)	.960 (.896–.985)	.959 (.895–.984)
Normal speed	.999 (.998–1.000)	.932 (.823–.974)	.946 (.860–.979)	.927 (.810–.972)
Fast speed	.971 (.930–.988)	.928 (.826–.970)	.975 (.940–.990)	.972 (.933–.988)
*Test-retest reliability instrumented treadmill*				
*0 % slope*				
Slow speed	.995 (.988–.998)	.945 (.868–.977)	.955 (.892–.981)	.944 (.866–.977)
Normal speed	.990 (.977–.996)	.960 (.904–.983)	.976 (.941–.990)	.982 (.956–.992)
Fast speed	.991 (.978–.996)	.956 (.895–.982)	.988 (.971–.995)	.984 (.960–.993)
*15 % slope*				
Slow speed	.996 (.991–.998)	.908 (.779–.962)	.935 (.843–.973)	.930 (.831–.971)
Normal speed	.993 (.983–.997)	.928 (.827–.970)	.946 (.870–.978)	.925 (.819–.969)
Fast speed	.996 (.991–.998)	.962 (.909–.984)	.972 (.933–.988)	.970 (.927–.987)

ICCs of data measured using both devices were used to determine validity. ICCs of data measured on the two separate days were used to determine the test-retest reliability of each system

Normal speed – habitual walking speed; slow speed – 85 % normal walking speed; fast speed – 115 % normal walking speed

**Table 4:** Root means square (RMS) errors of the difference between values measured with the RehaGait® system and the instrumented treadmill for speed, stride length, cadence and stride time for each slope and walking on day 1 and day 2

Condition	Speed (m/s)	Stride length (m)	Cadence (steps/min)	Stride time (s)
*Day 1*				
*0 % slope*				
Slow speed	0.069	0.069	0.086	0.0037
Normal speed	0.078	0.064	0.093	0.0035
Fast speed	0.125	0.087	0.102	0.0028
*15 % slope*				
Slow speed	0.187	0.088	0.072	0.0036
Normal speed	0.075	0.075	0.085	0.0022
Fast speed	0.127	0.112	0.087	0.0034
*Day 2*				
*0 % slope*				
Slow speed	0.080	0.846	0.087	0.0030
Normal speed	0.084	0.061	0.087	0.0028
Fast speed	0.071	0.128	0.092	0.0023
*15 % slope*				
Slow speed	0.072	0.101	0.067	0.0028
Normal speed	0.083	0.090	0.073	0.0026
fast speed	0.090	0.095	0.110	0.0032

### Test-retest reliability

No significant main effect for time was observed for any gait characteristics (speed: *F* = 1.37, *P* = .267; stride length: *F* <0.01, *P* = .998; cadence: *F* = 1.58, *P* = .233; stride time: *F* = 1.31, *P* = .275). Both devices had excellent reliability for all gait characteristics except good reliability for the RehaGait® for stride length at normal and fast speed at 0 % slope and at slow speed at 15 % slope (Table 3). The limits of agreement for repeated measurements of stride length, cadence and stride time with the RehaGait® were larger at 15 % slope than at 0 % slope. There was no systematic difference in RMS errors of the difference in gait parameters between the two systems between day 1 and day 2 (Table 1).

Main effects for speed and slope were observed for stride length (speed: F = 167.8, *P* < .001; slope: *F* = 16.4, *P* = .002), cadence (speed: *F* = 134.1, *P* < .001; slope: *F* = 10.8, *P* = .006) and stride time (speed: *F* = 112.7, *P* < .001; slope: *F* = 10.6, *P* = .007; Table 4).

## Discussion

The purpose of this study was to assess the validity of the RehaGait® with a stationary treadmill and the reliability of both systems at different velocities and slopes. We found good to excellent validity for stride length, cadence and stride time between the RehaGait® system and the instrumented treadmill in healthy younger adults. The RehaGait® system overestimated stride length (+2.7 %) and stride time (+0.8 %) and underestimate cadence (−1.5 %) with small to moderate effect sizes for all speeds and slopes. ICCs were slightly lower at slow compared to normal and fast walking speeds which is in agreement with previous findings [12]. Low day-to-day variability indicates good to excellent reliability of the RehaGait® system. Larger limits of agreement for walking at 15 % slope suggests that uphill walking may influence the reliability of the RehaGait® system.

Although significant differences in all gait characteristics were found between the RehaGait® and the instrumented treadmill, these differences were smaller than differences in these gait characteristics between other body-worn gyroscope based sensors and the GAITRite® system [28]. In our study, the average difference in stride length was less than 5 cm for walking at normal speed at 0 % slope compared to almost 8 cm reported by Greene et al. [28]. The latter study assessed spatiotemporal gait characteristics at a much greater range of walking speeds (0.89–1.72 m/s) than our study (0.91–1.30 m/s), and the normal speed in the study by Greene et al. corresponds to the fast speed in our study. While different environmental factors affect gait, walking speed is mainly modulated by altering stride length and only small changes in cadence [29]. Differences in spatiotemporal gait characteristics between accelerometer based gait analysis and the GAITRite® of less than 0.02 m/s walking speed, 1 cm step length and 2 ms step time in older adults have been reported [30]. The agreement between data obtained with the RehaGait and the instrumented treadmill was better by a factor of 10 for stride time and worse by a factor of 2 than that of other wearable technology [12]. Hence, the RehaGait® can be used to assess spatiotemporal gait characteristics of level treadmill walking with sufficient accuracy, although the agreement of temporal parameters is better than that of spatial and spatiotemporal parameters shown by the RMS errors. However, we cannot elucidate conclusively if the greater agreement between data measured using RehaGait® and the instrumented treadmill compared to that of other portable systems can be attributed to improved algorithms or technology or to differences in methodology.

To date, only few studies have investigated the effect of inclined walking at different speeds on basic spatiotemporal gait characteristics. For instance, Leroux et al. [23] reported that postural adaptations to increasing walking surface slope are accompanied by gradual increases in stride length as the uphill slope becomes steeper. In our study, we also observed greater stride length when walking on a 15 % slope compared to level walking. Interestingly, the increases in stride length detected by the RehaGait® were greater than those detected by the instrumented treadmill resulting in lower validity for walking on 15 % slope than on 0 % slope for the RehaGait®. One possible explanation for this discrepancy is that – albeit not recorded in this study – the foot striking pattern may change when walking uphill potentially affecting the accuracy of identifying gait events by both systems. For instance, foot strike may transition from foot strike to midfoot strike when walking uphill hence potentially altering acceleration (inertial sensor) and pressure (instrumented treadmill) patterns. Hence, such changes in gait mechanics may affect the accuracy of identifying foot strike for both systems hence potentially affecting spatial and spatiotemporal gait parameters. Our results confirm previously reported [31] decreases in cadence with increasing uphill slope only for slow speeds. However, in our study speed was kept constant for walking at both slopes, and hence participants were not able to freely adjust their gait patterns to the changing environment.

Other systems comprising inertial sensors mounted to the foot have been shown to provide accurate estimates of walking speed and incline for walking at a range of speeds and inclines, respectively [32]. The purpose of our study was to test the system validity of the RehaGait® system, and hence we only tested two inclines commonly used in clinical environments [20, 21]. The fact that walking speed recorded by the RehaGait® adequately corresponded to the speed set on the treadmill tachometer at flat and inclined slope emphasizes the validity of this portable gait analysis system for measuring walking speed for treadmill walking. Moreover, the large discrepancy in walking speed set on the treadmill tachometer and that indirectly calculated from the built in pressure mat suggests that the tachometer speed of the instrumented treadmill is more reliable than the calculated speed.

Several factors may have contributed to the differences in gait characteristics between RehaGait® and the instrumented treadmill. First, the two systems measure two different quantities. The RehaGait® measures the acceleration and angular velocity of the foot while the instrumented treadmill measures the pressure distribution under the foot. Based on these different quantities, specific algorithms are employed to calculate gait events that may be differently affected by factors such as foot placement. Slight differences in these definitions may cause systematic differences in gait characteristics between the two systems. The double integration of the acceleration signal may affect the calculation of spatiotemporal gait characteristics such as walking speed and stride length and hence explain the slightly lower validity for these characteristics. However, because potential drift is offset during zero acceleration phases of the sensor, this effect is expected to be minimal. Moreover, stride length assessed by the instrumented treadmill was calculated as the distance between two initial heel pressure points of alternate sides. Hence, even small step-to-step variability in foot placement (heel-strike versus midfoot-strike) would contribute to inaccuracies in calculated stride length and hence also in calculated walking speed explaining the discrepancy between latter and the tachometer speed.

In this study, we compared gait characteristics between the RehaGait® and an instrumented treadmill. The advantage of the RehaGait® over laboratory based 3days gait analysis systems is that the RehaGait® can be used to assess gait in free-living conditions. While treadmill walking differs from overground walking [33], a previous study [6] has shown that body worn sensor technology provide valid and reliable gait characteristics only for gait that is performed over walking distances exceeding 20 m. In our study, we measured gait data for approximately 200 strides, and a validation for gait characteristics for such distances can only be performed using a treadmill. Moreover, the use of a treadmill for validating the RehaGait® enabled us to control the walking speeds both for level walking and for walking on an incline. In this study, we only included healthy younger subjects and hence cannot make a statement regarding validity of the RehaGait® in other populations such as older adults or people with pathologies.

Overall, our results further support the use of inertial sensor based gait analysis system with associated advantages of facilitating cost and time efficient assessment of gait patterns. However, the applicability of such systems depends on the clinical and research question because only selected gait parameters can be assessed. For instance, current inertial sensor based systems cannot measure stride width, a parameter that has been identified as being important for predicting fall risk [2, 3]. Future research is warranted to elucidate other surrogate measures for risk of falls and of neuromuscular and musculoskeletal conditions that can be assessed using these novel sensors.

## Conclusions

RehaGait® is a commercial mobile system that specializes on measuring gait characteristics in field settings enabling gait data collection within less than 10 min. We showed good to excellent validity for walking speed, stride length, cadence and stride time between the RehaGait® system and the instrumented treadmill in a convenience sample of healthy younger adults with good to excellent reliability. Larger limits of agreement for walking at 15 % slope than at 0 % slope suggests that walking slope may influence the reliability of the RehaGait® system. Spatiotemporal gait information obtained with this system can be used for functional evaluations of patient populations augmenting clinical assessments for treatment planning and evaluation.
